# Polycomb Protein SCML2 Associates with USP7 and Counteracts Histone H2A Ubiquitination in the XY Chromatin during Male Meiosis

**DOI:** 10.1371/journal.pgen.1004954

**Published:** 2015-01-29

**Authors:** Mengcheng Luo, Jian Zhou, N. Adrian Leu, Carla M. Abreu, Jianle Wang, Montserrat C. Anguera, Dirk G. de Rooij, Maria Jasin, P. Jeremy Wang

**Affiliations:** 1 Department of Animal Biology, School of Veterinary Medicine, University of Pennsylvania, Philadelphia, Pennsylvania, United States of America; 2 Developmental Biology Program, Memorial Sloan-Kettering Cancer Center, New York, New York, United States of America; 3 Center for Reproductive Medicine, Academic Medical Center, University of Amsterdam, Amsterdam, The Netherlands; Cornell University, UNITED STATES

## Abstract

Polycomb group proteins mediate transcriptional silencing in diverse developmental processes. Sex chromosomes undergo chromosome-wide transcription silencing during male meiosis. Here we report that mouse SCML2 (Sex comb on midleg-like 2), an X chromosome-encoded polycomb protein, is specifically expressed in germ cells, including spermatogonia, spermatocytes, and round spermatids. SCML2 associates with phosphorylated H2AX and localizes to the XY body in spermatocytes. Loss of SCML2 in mice causes defective spermatogenesis, resulting in sharply reduced sperm production. SCML2 interacts with and recruits a deubiquitinase, USP7, to the XY body in spermatocytes. In the absence of SCML2, USP7 fails to accumulate on the XY body, whereas H2A monoubiquitination is dramatically augmented in the XY chromatin. Our results demonstrate that the SCML2/USP7 complex constitutes a novel molecular pathway in modulating the epigenetic state of sex chromosomes during male meiosis.

## Author Summary

Chromatin-modifying enzymes regulate the chromatin state during development and disease. Polycomb group proteins control the expression of homeotic genes in developmental patterning by catalyzing post-translational modifications of histones—core protein components of the chromatin. Most studies have focused on widely expressed polycomb proteins. However, the tissue-specific roles of polycomb proteins are poorly understood. Here we report functional studies of a testis-specific polycomb protein—SCML2. The *Scml2* gene maps to the X chromosome. Intriguingly, the SCML2 protein localizes specifically to the XY chromatin in germ cells during male meiosis, which undergoes chromosome-wide transcriptional silencing. Disruption of *Scml2* causes defects in spermatogenesis in mice. SCML2 associates with phosphorylated H2AX and a deubiquitinase, USP7. While localization of phosphorylated H2AX to the XY chromatin is SCML2-independent, USP7 localizes to the XY chromatin in an SCML2-dependent manner. Loss of SCML2 results in accumulation of H2A monoubiquitination in the XY chromatin in spermatocytes. These functional studies of SCML2 uncover a new molecular pathway that regulates H2A ubiquitination on the sex chromosomes during male meiosis.

## Introduction

Polycomb group (PcG) proteins are key epigenetic factors in maintaining transcriptional silencing during development in higher eukaryotes [1]. PcG proteins form chromatin-modifying complexes, notably polycomb repressive complex 1 (PRC1) and PRC2. PRC2 mediates trimethylation of histone H3 on lysine 27 (H3K27me3) through its methyl transferase activity. Recruitment of PRC1 to the chromatin involves its binding to H3K27me3, but in some instances, is PRC2/H3K27me3-independent [2]. PRC1 mediates monoubiquitination of histone H2A at lysine 119 through the ubiquitin E3 ligase activity of one of its components—RNF2 [3]. H2A ubiquitination is linked with transcriptional silencing and X-inactivation [3, 4]. Self-ubiquitination of RNF2 is required for its ubiquitin E3 ligase activity [5]. USP7, a deubiquitinating enzyme, directly deubiquitinates RNF2 [5]. These studies demonstrate the intricacy in the regulation of polycomb protein-mediated silencing.

*Drosophila* SCM (Sex comb on midleg) is a poorly characterized polycomb protein and does not appear to be a core component of PRC1 or PRC2 [6–8]. SCM contains two malignant brain tumor (MBT) repeats close to its N-terminus, a DUF3588 domain, and a C-terminal sterile alpha motif (SAM). In mammals, there are at least four SCM homologues: SCMH1, SCML1, SCML2 and SFMBT. Based on the crystal structure, the MBT repeat of SCML2 is capable of binding to peptides with mono-methylated lysine [9, 10]. A recent NMR study shows that the DUF3588 domain (also called Scm-like embedded domain—SLED) binds to DNA in a sequence-specific manner [11]. The SAM domain mediates the association of SCM proteins with PRC1 [7]. SCMH1 is a substoichiometric constituent of mammalian PRC1 [7].

During male meiosis, sex chromosomes form the so-called sex body (XY body) and undergo chromosome-wide transcriptional silencing, a phenomenon termed MSCI (meiotic sex chromatin inactivation) [12–14]. Phosphorylated H2AX (γH2AX) is required for formation of the XY body and thus MSCI [12, 15]. During formation of the XY body, ATR phosphorylates H2AX and MDC1 binds to γH2AX to direct sex chromosome-wide silencing [16, 17]. Transcriptional silencing of sex chromosomes persists into the post-meiotic stage [18, 19]. Formation of the XY body is accompanied by various histone modifications such as ubiquitination and methylation [20].

Mouse SCMH1 and core components of PRC1 are excluded from the XY body in the pachytene stage of male meiosis. Disruption of *Scmh1* causes sterility in half of *Scmh1^-/-^* male mice [20]. Testes from the sterile *Scmh1* ^-/-^ mice exhibit apoptosis of late pachytene spermatocytes and lack post-meiotic spermatids. Genetic studies suggest that SCMH1 functions in the epigenetic modifications of the XY chromatin during spermatogenesis by excluding the PRC1 complex [20].

**Figure 1:**
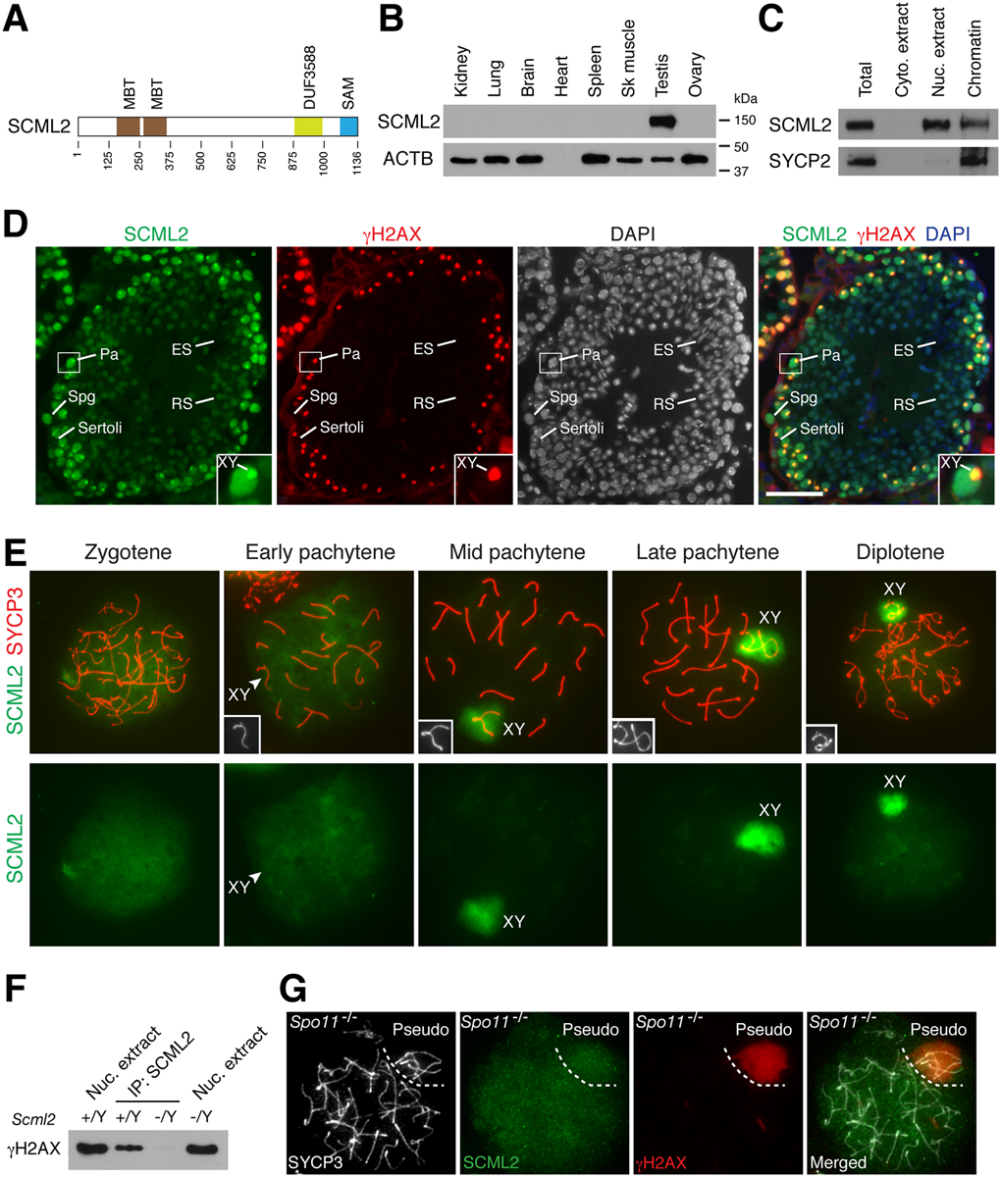
Expression and localization of SCML2 during mouse spermatogenesis. (A) Domain structure of mouse SCML2 protein (XP_006528733.1). MBT, malignant brain tumor repeat; DUF3588, also called Scm-like embedded domain (SLED) [11]; SAM, sterile alpha motif. (B) Western blot analysis of SCML2 in adult mouse tissues. ACTB serves as a control. (C) Western blot analysis of SCML2 on cytoplasmic extract, nuclear extract, and chromatin from postnatal day 20 testis. SYCP2, a synaptonemal complex protein, serves as a control [44]. (D) Expression and localization of SCML2 in male germ cells. Testis sections from 2-month-old mice were immunostained with SCML2 and γH2AX antibodies. Nuclei were stained with DAPI. The inset is an enlarged view of a pachytene spermatocyte with the XY body indicated. Abbreviations: Sertoli, Sertoli cells; Spg, spermatogonium; Pa, pachytene spermatocyte; RS, round spermatid; ES, elongated spermatid. Scale bar, 50 μm. (E) Localization of SCML2 to the XY body in wild type spermatocytes. Spread nuclei were immunostained with anti-SCML2 and anti-SYCP3 antibodies. SCML2 staining alone are shown in bottom panels. XY chromosomes are shown in insets. (F) Co-immunoprecipitation of SCML2 and γH2AX from wild type (*Scml2* ^+/Y^) testicular nuclear extracts. *Scml2* ^-/Y^ testis was used as a negative control. (G) Lack of preferential accumulation of SCML2 in the pseudo sex body in *Spo11* ^-/-^ zygotene-like spermatocytes.

We previously identified SCML2 as a meiotic chromatin-associated protein [21]. SCML2 is encoded by the X chromosome [22]. Like SCM, SCML2 consists of two MBT repeats, DUF3588, and a SAM motif (Fig. 1A). Structural studies of the MBT and DUF35588 domains in SCML2 support that it binds to chromatin [9–11]. Human *SCML2* gene encodes two protein isoforms: SCML2A (chromatin-bound) and SCML2B (nucleoplasmic) [23]. In cultured immortal or cancer cells, human SCML2A interacts with PRC1 and binds to non-coding RNAs [24], whereas human SCML2B regulates the cell cycle by binding to CDK2 [23]. Here we report genetic and functional studies of SCML2 in mice and demonstrate that SCML2 regulates the epigenetic state of sex chromosomes during male meiosis.

## Results

### SCML2 Is a Germ Cell-Specific Chromatin-Associated Protein

Western blot analysis showed that the mouse SCML2 protein was expressed in testes but not in adult ovary or somatic tissues (Fig. 1B). SCML2 protein was detectable in fetal ovary, but at a much lower level than in postnatal testis (S1A Fig.). The apparent molecular weight of mouse SCML2 is 150 kDa (Fig. 1B). Western blot analysis on cellular fractions revealed that SCML2 was associated with chromatin in testis (Fig. 1C), in consistence with its identification as one of the meiotic chromatin-associated proteins in our proteomics screen [21]. In addition, SCML2 was detected in the soluble nuclear extract but not in the cytoplasmic extract (Fig. 1C). Human *SCML2* gene encodes two protein isoforms (SCML2A and SCML2B) [23]. In contrast, only a single SCML2 protein band was detected in mice (Fig. 1B, C). Immunolocalization studies revealed that the SCML2 protein was present in germ cells but absent in testicular somatic cells such as Sertoli cells (Fig. 1D). SCML2 localized exclusively to the nucleus. SCML2 was abundantly expressed in spermatogonia and spermatocytes, and was present in round spermatids at a lower level. However, SCML2 was undetectable in elongated spermatids (Fig. 1D). These results demonstrate that SCML2 is a nuclear protein with expression in spermatogonia through round spermatids in the testis.

### SCML2 Associates with γH2AX in the Sex Body in Spermatocytes

While it localized throughout the nucleus, SCML2 concentrated on the XY body in spermatocytes and colocalized with γH2AX, which is known to coat the XY chromatin during male meiosis (Fig. 1D). To determine the timing of appearance of SCML2 on the XY body, we performed surface nuclear spread analysis of spermatocytes (Fig. 1E). SCML2 appeared throughout the chromatin of zygotene spermatocytes. SCML2 did not concentrate on the XY body in 84% of early pachytene spermatocytes (n = 273) but localized to the XY body in 95% of mid-to-late pachytene spermatocytes (n = 381) and 95% of diplotene spermatocytes (n = 131). The localization pattern of SCML2 in pachytene spermatocytes suggests that SCML2 may function in the maintenance instead of initiation of MSCI. The localization of SCML2 to the XY body was in stark contrast with that of another SCM protein—SCMH1, which is excluded from the XY body [20]. To test whether SCML2 forms a complex with γH2AX in vivo, we performed co-immunoprecipitation using nuclease-treated testicular nuclear extracts followed by Western blot analysis. This result showed that SCML2 is associated with γH2AX in testis in a DNA-independent manner (Fig. 1F).

*Spo11* is essential for DNA double strand break formation in meiotic recombination [25, 26]. In *Spo11* ^-/-^ spermatocytes, a sex body-like structure called the pseudo sex body forms due to extensive chromosome unsynapsis [27, 28]. We examined the localization of SCML2 in *Spo11* ^-/-^ spermatocytes. Pseudo sex body (γH2AX-positive) formed in 86 out of 192 *Spo11* ^-/-^ zygotene-like spermatocytes examined from three *Spo11* ^-/-^ mice (Fig. 1G). However, SCML2 did not accumulate in the pseudo sex body in any of *Spo11* ^-/-^ zygotene-like spermatocytes, suggesting that γH2AX is not sufficient for recruitment of SCML2 (Fig. 1G). Alternatively, the *Spo11* ^-/-^ zygotene-like spermatocytes might not have advanced to the mid-late pachytene stage.

### Defective Spermatogenesis in *Scml2* ^-/Y^ Mice

**Figure 2:**
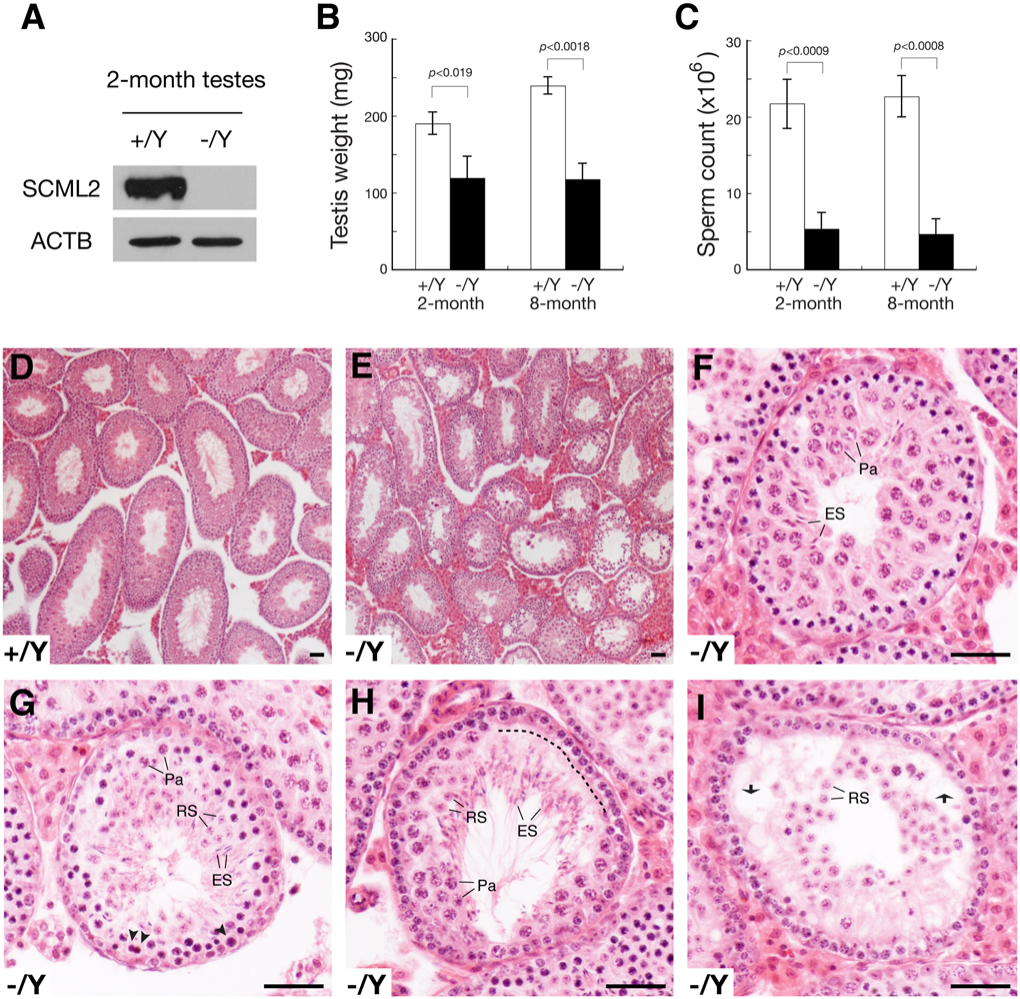
Defective spermatogenesis in *Scml2* ^-/Y^ mice. (A) Western blot analysis of wild type and *Scml2* ^-/Y^ testes. (B) Dramatic testis weight reduction (per pair of testes) in *Scml2* ^-/Y^ mice. (C) Sharp reduction in sperm count (per pair of cauda epididymides) in *Scml2* ^-/Y^ mice. The body weight of age-matched male mice was similar. Body weight at the 2-month age: wild type, 28.0 ± 1.5 g and *Scml2* ^-/Y^, 28.6 ± 1.0 g (n = 3 per genotype). Body weight at the 8-month age: wild type, 39.4 ± 4.7 g and *Scml2* ^-/Y^, 39.3 ± 5.5 g (n = 3 per genotype). (D) Histology of a 4-month-old wild-type testis at low magnification. (E-I) Variable spermatogenic defects in tubules from 4-month-old *Scml2* ^-/Y^ testes. Testis section is shown in low magnification (E). Spermatogenesis in some tubules (F) is relatively normal. Apparently apoptotic spermatocytes are indicated by arrowheads (G). A layer of lost spermatocytes is marked by a dashed line (H). Some tubules (I) exhibit nearly complete depletion of pachytene spermatocytes. Vacuoles are indicated by arrows (I). Abbreviations (F-I): Pa, pachytene spermatocytes; RS, round spermatids; ES, elongated spermatids. Scale bars, 50 μm.

To determine the role of *Scml2* in spermatogenesis, we generated a floxed allele (*Scml2* ^fl^) in mice using homologous recombination in embryonic stem (ES) cells. In the targeted allele, exon 11 was flanked by *loxP* sites in introns (S2 Fig.). To disrupt the *Scml2* gene, *Scml2* ^fl/Y^ mice were bred with *Actb*-Cre mice, in which Cre is widely expressed in the embryo [29]. Deletion of exon 11 was expected to cause a frameshift in the resulting mutant transcript. Western blotting analysis showed that the SCML2 protein was absent in the *Scml2* ^-/Y^ testes, indicating that our *Scml2* mutant allele is null (Fig. 2A). Because the SCML2 antigen used for antibody production partially overlapped with the protein region encoded by the deleted exon, we could not exclude the possibility that our antibody failed to recognize a truncated SCML2 protein in the mutant testis. Nevertheless, lack of SCML2 in *Scml2* mutant testicular sections by immunofluorescence confirmed the specificity of our anti-SCML2 antibody (S3 Fig.).

The *Scml2* ^-/Y^ males and *Scml2* ^-/-^ females appeared to be grossly healthy. The body weight of age-matched adult mice was similar between wild type and mutant. Breeding of *Scml2* ^+/-^ females with wild type males yielded a normal Mendelian ratio of offspring (*Scml2* ^+/Y^, 57; *Scml2* ^-/Y^, 60), suggesting that inactivation of *Scml2* does not cause lethality. Histological analysis of adult *Scml2* ^-/-^ ovaries revealed no defects in oogenesis, which is consistent with the lack of SCML2 in adult ovaries (Fig. 1B), an extremely low level of SCML2 in fetal ovaries (S1A Fig.), and lack of SCML2 in spread nuclei of fetal pachytene oocytes (S1B Fig.). We tested the fertility of seven 4-month-old *Scml2* ^-/Y^ males and three wild type littermate males by housing one male with two wild type females for six weeks. During the 6-week mating period, all three wild type males sired at least one litter (8.4 ± 2.2 pups/litter, n = 5). In contrast, only two *Scml2* ^-/Y^ males sired one litter each (6 and 7 pups/litter) and the remaining five mutant males did not produce any offspring, suggesting variable fertility defects in *Scml2* ^-/Y^ males. The weight of adult *Scml2* ^-/Y^ testes was lower than that of the wild type (Fig. 2B). The sperm count of adult *Scml2* ^-/Y^ males was 75% less than that of the wild type (Fig. 2C). While wild type seminiferous tubules from adult testes contained a full spectrum of spermatogenic cells (Fig. 2D), the *Scml2* ^-/Y^ testes exhibited variable spermatogenic defects among the seminiferous tubules (Fig. 2E). Some mutant tubules had relatively normal spermatogenesis (Fig. 2E, F). A cohort of pachytene spermatocytes apparently underwent apoptosis in some mutant tubules (Fig. 2G). In part of this mutant tubule, a layer of pachytene spermatocytes was missing, possibly caused by apoptosis (Fig. 2H). Some tubules lacked the entire layer of pachytene spermatocytes and developed vacuoles (Fig. 2I). These genetic studies suggest that *Scml2* plays an important but non-essential role in spermatogenesis.

During meiosis, homologous chromosomes undergo synapsis and recombination. We monitored these two meiotic processes in *Scml2* ^-/Y^ males. Surface nuclear spread analysis of the synaptonemal complex did not reveal defects in chromosomal synapsis in *Scml2* ^-/Y^ pachytene spermatocytes (S4A Fig.). While spermatocytes at all meiotic stages were present in *Scml2* ^-/Y^ testes, the percentage of diplotene spermatocytes was reduced in the mutant (S4B Fig.). MLH1 marks the site of meiotic crossovers [30, 31]. The number of MLH1 foci in *Scml2* ^-/Y^ pachytene spermatocytes (22.3±1.67, n = 62) was similar to that in wild type spermatocytes (22.0±1.66, n = 59), suggesting that crossover formation is not affected by loss of SCML2.

### Apoptosis of Pachytene Spermatocytes in *Scml2* ^-/Y^ Testes

**Figure 3:**
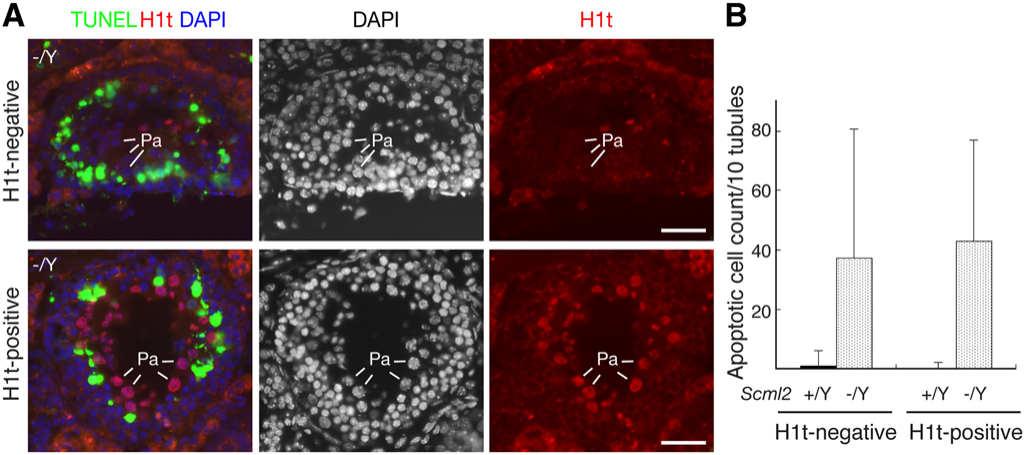
Apoptosis of spermatocytes in adult *Scml2* ^-/Y^ testes. Based on the expression of histone H1t in pachytene spermatocytes, seminiferous tubules were divided into two groups: H1t-negative (early pachytene stage) and H1t-positive (mid-late pachytene stage). The number of TUNEL-positive cells per tubule cross-section was counted. (A) Apoptosis in both H1t-negative and H1t-positive *Scml2* ^-/Y^ seminiferous tubules. Apoptotic cells presumably corresponded to pachytene spermatocytes. Pa: pachytene spermatocytes. Scale bars, 25 μm. (B) Count of apoptotic cells. 100 tubules from adult *Scml2* ^-/Y^ testes and 239 tubules from wild type testes were analyzed.

Spermatocytes appeared to undergo apoptosis in *Scml2* ^-/Y^ testes (Fig. 2G). To determine at what stage germ cells die, we performed TUNEL assay on frozen testicular sections (Fig. 3A). For this analysis, we used histone H1t as a marker to divide pachytene spermatocytes into two groups: early pachytene (H1t-negative) and mid-to-late pachytene (H1t-positive). H1t is not expressed in early pachytene spermatocytes, appears in mid pachytene spermatocytes, and is abundant in late pachytene spermatocytes [32]. In wild type seminiferous tubules from adult testes, apoptotic cells were rare. In contrast, the number of apoptotic cells increased dramatically in *Scml2* ^-/Y^ tubules (Fig. 3B). Furthermore, this increase occurred in both H1t-negative and H1t-positive tubules, suggesting that both early and mid-to-late pachytene spermatocytes undergo apoptosis in *Scml2* ^-/Y^ males.

### MSCI Is Not Affected in *Scml2*-Deficient Spermatocytes

We next examined the formation of the XY body in *Scml2* ^-/Y^ spermatocytes. Like γH2AX, SUMO1 (small ubiquitin-like modifier 1) localizes to the XY body [33–35]. We found that both γH2AX and SUMO1 were present in the XY body in *Scml2*-deficient pachytene spermatocytes (S5 Fig.). We further examined their localization in early stages such as leptotene and zygotene. γH2AX was abundant throughout the nuclei of leptotene and zygotene spermatocytes from both wild type and *Scml2* ^-/Y^ males due to the programmed double strand break formation during meiosis (S5A Fig.). As in wild type [33], SUMO1 was not detected in the spread nuclei of *Scml2*-deficient leptotene and zygotene spermatocytes (S5B Fig.). These results show that the localization of γH2AX and SUMO1 is independent of SCML2.

To determine the effect of loss of SCML2 on the expression of X-linked genes, we examined the expression of an X-encoded germ cell-specific protein PRAMEL3 by immunofluorescence. As previously reported [36], PRAMEL3 was expressed in early spermatocytes, absent in pachytene spermatocytes, but reactivated in post-meiotic round spermatids (S6A Fig.). The expression pattern of the PRAMEL3 protein in *Scml2* ^-/Y^ tubules was unchanged (S6A Fig.), suggesting that inactivation of SCML2 did not cause a complete failure in transcriptional silencing of the X chromosome during male meiosis. To further examine MSCI, we performed Cot-1 RNA FISH. Cot-1 DNA is enriched for repetitive sequences, which are frequently present in introns and 3’UTRs of pre-mRNA transcripts, and thus hybridizes with nascent transcripts [12, 37]. As in the wild type spermatocytes, the XY body was positive for γH2AX and resided in a Cot-1 negative region in *Scml2* ^-/Y^ spermatocytes, indicative of transcriptional silence (S6B Fig.). These results suggest that MSCI is intact in *Scml2* ^-/Y^ spermatocytes.

### SCML2 Forms a Complex with USP7 *In Vivo*

**Figure 4:**
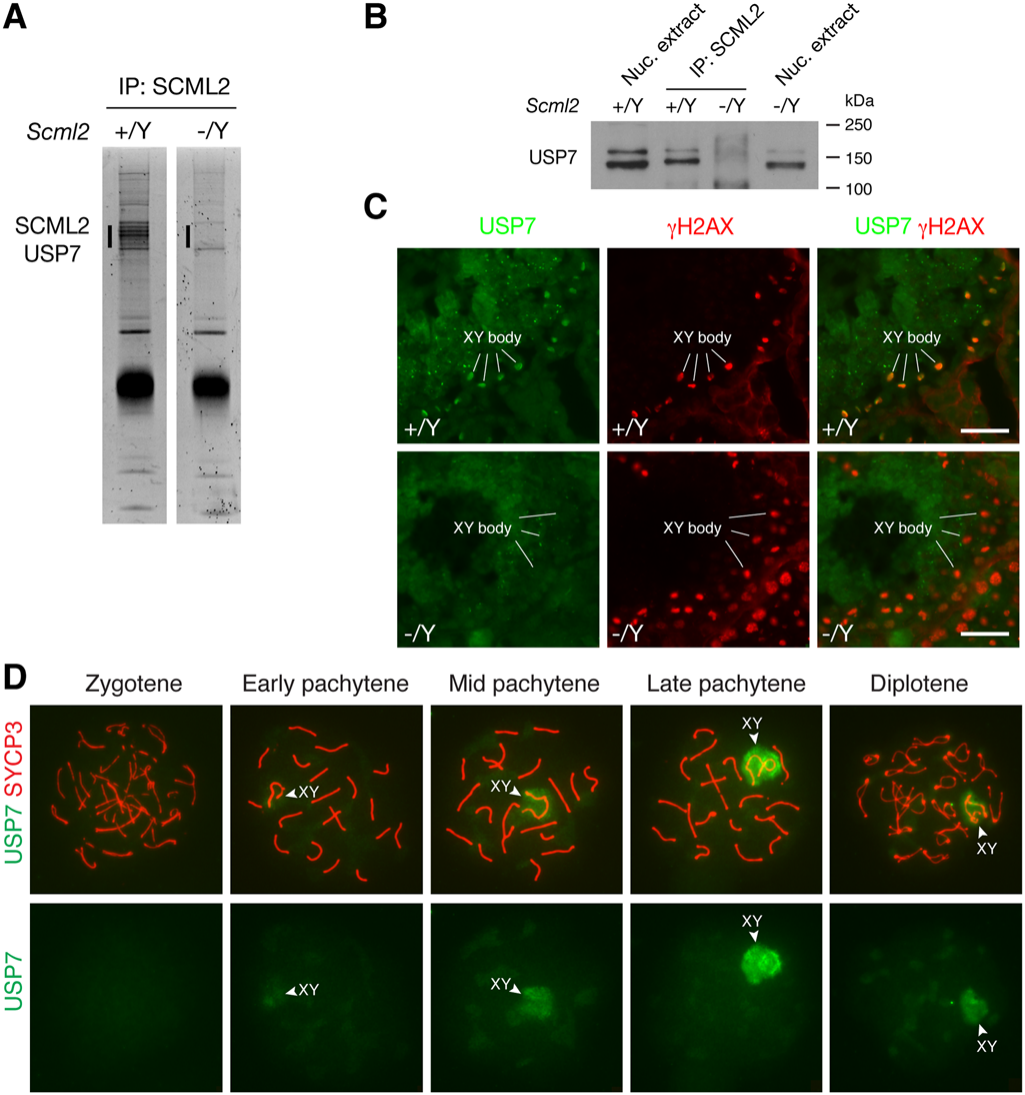
SCML2 associates with USP7 in the XY body. (A) Identification of SCML2-associated proteins from 20-day testes by immunoprecipitation (IP) and mass spectrometry. The extra bands (vertical line) in the wild type IP and the corresponding region (vertical line) in the *Scml2* ^-/Y^ IP were subjected to mass spectrometry. (B) Co-immunoprecipitation of SCML2 with USP7 in testes. Nuclear protein extracts prepared from 20-day testes were used. *Scml2* ^-/Y^ testes were used as a negative control. Note that the USP7 antibody (Bethyl Laboratory) recognizes two bands with the lower band being more abundant. Both USP7 bands were co-immunoprecipitated with SCML2. However, the nature of the two USP7 isoforms is unknown. (C) Immunolocalization of USP7 and γH2AX in wild type and *Scml2* ^-/Y^ seminiferous tubules from 8-week-old mice. Testicular frozen sections were used for double immunostaining. Scale bars, 25 μm. (D) Localization of USP7 to the XY body in wild type spermatocytes. Spread nuclei from prophase I spermatocytes (from zygotene to diplotene stages) were immunostained with anti-USP7 and anti-SYCP3 antibodies. USP7 localization alone is shown in bottom panels.

To search for additional SCML2-interacting partners, we performed immunoprecipitation using testicular nuclear extracts with our anti-SCML2 antibodies. Extra bands were present in the immunoprecipitated proteins from the wild type compared to the *Scml2* ^-/Y^ testes (Fig. 4A). Mass spectrometry analysis identified these proteins as SCML2 and USP7. USP7 is a widely expressed deubiquitinating enzyme involved in a variety of processes such as DNA replication, apoptosis, and tumorigenesis [5]. Immunoprecipitation followed by Western blot analysis confirmed the association of SCML2 with USP7 in the wild type testis (Fig. 4B). As expected, USP7 was not co-immunoprecipitated from *Scml2* ^-/Y^ testicular extract (Fig. 4B).

We next examined the expression of USP7 during spermatogenesis by immunofluorescence. While it was present in all cells in the testicular tubules, it preferentially localized to the XY body in spermatocytes, which was γH2AX-positive (Fig. 4C). Furthermore, immunolocalization analysis revealed that USP7 was absent in the XY body in *Scml2*-deficient spermatocytes, while γH2AX was present in the XY body in the mutant cells (Fig. 4C).

Nuclear spread analysis of spermatocytes confirmed the localization of USP7 to the XY body in pachytene spermatocytes, demonstrating that USP7 is a novel component of the XY body (Fig. 4D). Notably, USP7 was barely detectable in the XY body in 98% of early pachytene spermatocytes (n = 168). In contrast, USP7 localized prominently to the XY body in 86% of mid-late pachytene spermatocytes (n = 321) and 59% of diplotene spermatocytes (n = 288) (Fig. 4D). The timing of USP7 localization to the XY body resembled that of SCML2 (Fig. 1E). In conclusion, SCML2 associates with USP7 and USP7 localizes to the XY body in an SCML2-dependent manner during male meiosis.

### Increased H2A Monoubiquitination in the XY Body in the Absence of SCML2

**Figure 5:**
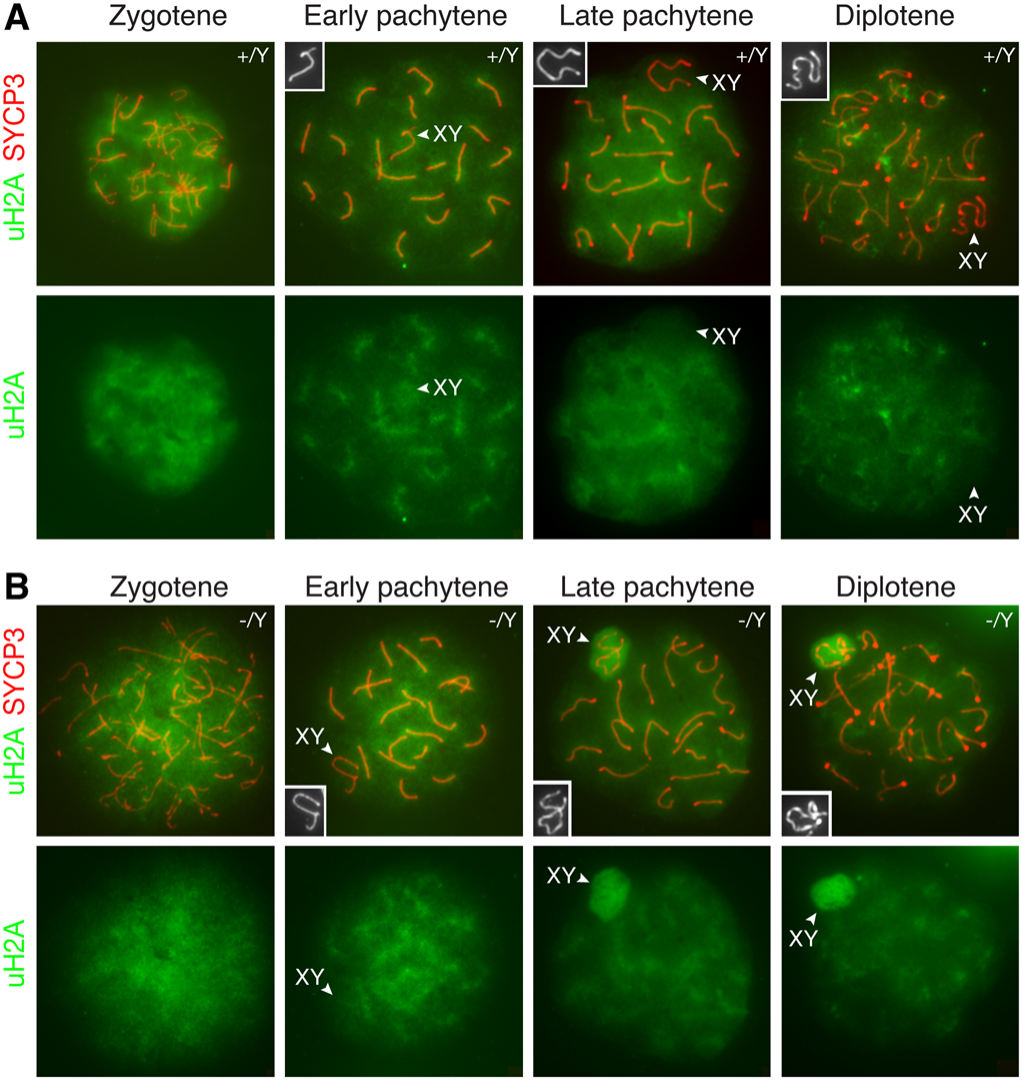
Increased H2A monoubiquitination in the XY body in *Scml2*-deficeint pachytene and diplotene spermatocytes. Distribution of monoubiquitinated H2A (uH2A) in wild type (A) and *Scml2* ^-/Y^ (B) prophase I spermatocytes were examined by immunostaining of spread nuclei with anti-SYCP3 (red) and anti-uH2A (green) antibodies. uH2A staining alone is shown in the bottom panels. The XY bodies are indicated by arrowheads and shown in the insets. Notably, uH2A localizes to and radiates from the synaptonemal complexes in wild type and *Scml2* ^-/Y^ pachytene spermatocytes.

PRC1 ubiquitinates histone H2A at lysine 119, resulting in monoubiquitinated H2A (uH2A) [3]. It is known that PRC1 components are excluded from the XY body in pachytene spermatocytes [20]. We used a monoclonal antibody that is specific for monoubiquitinated H2A at lysine 119. As expected, uH2A was not enriched in the XY body in wild type pachytene and diplotene spermatocytes (Fig. 5A). Our result was different from previous studies that reported increased H2A ubiquitination in the XY body in wild type spermatocytes, which appeared to be RNF8-dependent [14, 38]. It was most likely that, in the previous studies, H2A modification detected was polyubiquitination rather than monoubiquitination [14, 38]. We next examined the localization of uH2A in *Scml2*-deficient spermatocytes (Fig. 5B). uH2A localized diffusely throughout the chromatin of mutant zygotene spermatocytes. uH2A in the XY body did not increase in 96% of early pachytene spermatocytes (n = 136) from the mutant mice. Interestingly, uH2A was highly upregulated in the XY body in 89% of mutant mid-late pachytene spermatocytes (n = 317) and 59% of mutant diplotene spermatocytes (n = 285). These data suggest that SCML2 counteracts H2A monoubiquitination in the XY chromatin during male meiosis.

## Discussion

Here we have delineated a novel molecular pathway in mediating H2A ubiquitination in the XY chromatin during male meiosis. In the XY body, SCML2 is associated with but functions downstream of or in parallel with γH2AX. SCML2 forms a complex with USP7 and recruits USP7 to the XY body. USP7 results in reduced H2A monoubiquitination in the XY chromatin. The effect of USP7 on deubiquitination of H2A is most likely indirect, since USP7 selectively deubiquitinates histone H2B, but not H2A [39]. PRC1 catalyzes specifically monoubiquitination of H2A with RNF2 being the E3 ligase [3]. The activity of RNF2 is regulated by its ubiquitination state. Self-ubiquitination of RNF2 is required for its E3 ligase activity. USP7 inhibits the enzymatic activity of RNF2 through deubiquitination [5]. It is possible that in wild type spermatocytes, USP7 may directly deubiquitinate RNF2 and thus reduce H2A ubiquitination in the XY body. In the absence of SCML2, USP7 fails to localize to the XY body and thus RNF2 is expected to remain ubiquitinated and active, leading to increased H2A ubiquitination.

H2A ubiquitination is associated with transcriptional silencing [3, 4]. Lack of de-silencing of an X-linked gene *Pramel3* and exclusion of Cot-1 RNA signals from the XY body during the pachytene stage of meiosis in the *Scml2* ^-/Y^ testes suggest that MSCI is intact in the *Scml2* mutant. Human SCML2 is expressed in a number of somatic cell lines [23, 24]. Human SCML2 interacts with PRC1 and is recruited to the PRC target sites in the genome. Knockdown of *SCML2* in human cell lines results in mis-regulated expression of more than 500 somatic genes [24]. Therefore, the mouse SCML2 protein may modulate transcription during spermatogenesis. Even though chromosomal synapsis and meiotic recombination appeared normal in *Scml2* ^-/Y^ spermatocytes, both early and mid-late pachytene spermatocytes underwent increased apoptosis. Together with the known function of human SCML2 in gene expression [24], we postulate that apoptosis of *Scml2* ^-/Y^ spermatocytes might be caused by aberrant expression of somatically expressed genes in germ cells.

The *Scml2* gene is located on the X chromosome and thus is subjected to transcriptional silencing due to MSCI in pachytene spermatocytes [22]. Indeed, *Scml2* transcript is undetectable in pachytene spermatocytes by RNA FISH [40]. However, SCML2 protein is present in the XY body in spermatocytes. There are two possible explanations for this paradox. One possibility is that the *Scml2* gene is transcribed in the XY body at a level too low to be detected by RNA FISH. A more likely scenario is that the SCML2 protein, which is abundantly present throughout the nucleus in the zygotene stage, redistributes to the XY body at the pachytene stage.

Human SCML2A binds to a large repertoire of non-coding RNAs in somatic cell lines through its RNA-binding region and such RNA interactions direct SCML2A to the chromatin [24]. It would be imperative to address whether mouse SCML2 also binds to non-coding RNAs in testis and to examine the potential roles of RNAs in the dynamic localization of SCML2 on the chromatin in germ cells in future studies.

Recent studies have demonstrated that human SCML2 is expressed in a variety of somatic cells—immortal and cancer cell lines [23, 24]. However, we find that mouse SCML2 is specifically expressed in testes but not in any of the somatic tissues examined (Fig. 1B). It is known that a large number of germ cell-specific genes are expressed in human cancers and thus referred to as “cancer/testis” genes [41]. Consistent with its interaction with PRC1 and binding to chromatin, SCML2 has profound impact on the cellular activity when ectopically expressed in transformed or cancer cells [23, 24]. Therefore, *Scml2* should be considered as a cancer/testis gene and might be implicated in tumorigenesis.

## Materials and Methods

### Ethics Statement

Mice were maintained and used for experimentation according to the guidelines of the Institutional Animal Care and Use Committee of the University of Pennsylvania.

### Antibodies and Western Blot Analyses

The 6xHis-SCML2 (mouse, 147–321 aa) fusion protein was expressed in *E. coli* using the pQE-30 expression vector, purified with Ni-NTA resin, and used to immunize rabbits and guinea pigs at Cocalico Biologicals Inc., resulting in polyclonal antisera UP2323 (rabbit) and gp92 (guinea pig). Affinity purified SCML2 antibodies (1:1000) were used for immunofluorescence and Western blotting analyses. Other antibodies used were γH2AX (1:1000 or 1:200, Millipore), SYCP3 (1:200, Abcam), USP7 (1:200, Bethyl), and ACTB (1:7500, Sigma-Aldrich).

### Targeted Inactivation of the *Scml2* Gene

To generate the targeting construct, DNA fragments were amplified by high-fidelity PCR using an *Scml2*-containing mouse BAC clone (RPCI23–54I20). In the targeting construct, one *loxP* site was inserted in intron 10 and the floxed HyTK (hygromycin and thymidine kinase) double selection cassette was inserted in intron 11. All three *loxP* sites were in the same orientation (S2 Fig.). Hybrid V6.5 ES cells (C57BL/6 × 129/sv) were electroporated with linearized targeting construct (pUP113/*Cla*I) and were cultured in the presence of hygromycin B (120 μg/ml; Invitrogen) [42]. By screening 384 drug-resistant ES cell clones, we obtained 10 homologously targeted *Scml2* ^3lox^ clones. Two *Scml2* ^3lox^ ES cell clones (2D9 and 4E6) were electroporated with the pOG231 plasmid that expresses Cre. Cells were subjected to negative selection with gancyclovir (2 μM, Sigma) for removal of the HyTK cassette. Two *Scml2* ^fl^ ES cells were injected into B6C3F1 (Taconic) blastocysts. The *Scml2* ^fl^ allele was transmitted through the germline in chimeric mice. The *Scml2* ^fl^ mice were bred with *Actb*-Cre mice to delete the floxed exon ubiquitously, giving rise to *Scml2* ^+/-^ females [29]. All *Scml2* ^-/Y^ male mice used were offspring of *Scml2* ^+/-^ females. All offspring were genotyped by PCR. Wild type (370 bp) and floxed (560 bp, *Scml2* ^fl^) alleles were assayed by PCR with primers TGCCACAATTGGAGCTGTCT and AGATTCCTGAGGAGCTCTCA. The knockout (315 bp, *Scml2* ^-^) allele was assayed by PCR with the primers CCATGACACCTGGCCTACAA and AGATTCCTGAGGAGCTCTCA.

### Immunoprecipitation and Mass Spectrometry

Nuclear extracts prepared from 100 mg of 20-day wild type and *Scml2* ^-/Y^ testes were used for immunoprecipitation with affinity purified anti-SCML2 antibody. Nuclear extract was prepared using the NE-PER kit (Thermo Scientific). Immunoprecipitated proteins were run on a 8% SDS–PAGE gel and stained with SYPRO Ruby (Bio-Rad). The gel bands exclusive to the wild type testis sample were sent for protein identification by mass spectrometry at the PENN Proteomics Core Facility.

For co-immunoprecipitation experiments followed by Western blotting, 100 mg of 20-day testes (wild type or *Scml2* ^−/Y^) were homogenized in 1 ml lysis buffer with 50 μM MG132. Benzonase (90 U/ml) was added to the lysate. The lysate was incubated on a rocking platform at room temperature for 2 hours. The nuclear extract was used for immunoprecipitation with anti-SCML2 antibodies followed by Western blotting with either anti-γH2AX or anti-USP7 antibodies.

### Histological, Surface-Spread, and Immunofluorescence Analysis

For histology, testes were fixed in Bouin’s solution, dehydrated, embedded in paraffin, and sectioned using a microtome. Sections were prepared and stained with hematoxylin and eosin. For immunofluorescence, testes were fixed in 4% paraformaldehyde at 4°C overnight, dehydrated, processed, and sectioned using a cryostat. TUNEL analysis and H1t immunofluorescence were performed as previously described [43]. For surface-spread analysis, spermatocytes from 20-day testes and oocytes from E17.5 fetal ovaries were used as previously described [21]. The following primary antibodies were used for immunostaining on spread nuclei and/or frozen sections: SYCP1 (Abcam), SYCP2 [44], SYCP3 (Santa Cruz), SYCP3 (Abcam), USP7 (Bethyl), uH2AK119 (Cell Signaling), γH2AX (Millipore), SUMO1 (Life Technologies), MLH1 (BD Biosciences), and histone H1t [32]. Early, mid, and late pachytene spermatocytes were distinguished by the morphology of XY chromosomal axis, intensity of synaptonemal complex staining, and the morphology of the synaptonemal complex (Fig. 1E). Early pachytene spermatocytes are characterized by relatively low intensity of synaptonemal complex staining, lack of increased accumulation of SYCP2 or SYCP3 at the ends of synaptonemal complexes, and an extended configuration of the XY chromosome. In contrast, the late pachytene stage of spermatocytes typically has prominent accumulation of SYCP2 and SYCP3 at the ends of synaptonemal complexes and the number 8-shaped XY axis. The mid pachytene stage falls between early pachytene and late pachytene stages, mostly with a U-shaped XY axis.

### Cot-1 RNA Fluorescence in Situ Hybridization (FISH)

Cot-1 RNA FISH was performed as described [45]. For combined RNA FISH/γH2AX immunostaining, RNA FISH was performed first, followed by immunostaining. Postnatal-day-27 wild type and *Scml2* ^-/Y^ testes were used. Permeabilization and fixation were performed directly on seminiferous tubules. Germ cells were mechanically dissociated with forceps and cytospun onto slides. Slides were dehydrated by sequential treatments with 80%, 90%, and 100% (vol/vol) ethanol. Slides were incubated with FITC-labeled mouse Cot-1 DNA probe (Life Technologies) in a humidified chamber overnight at 42°C. Slides were washed and processed for immunostaining with γH2AX antibody and a secondary antibody. Digital images were acquired with the Nikon ECLIPSE Ni microscope with a 100× oil immersion lens using the NIS-Elements version 4.2 software (Nikon).

## Supporting Information

S1 Fig Expression analysis of SCML2 in the embryonic ovaries. (A) Western blot analysis of SCML2 in embryonic day 17.5 (E17.5) ovaries. In E17.5 ovaries, most oocytes are at the pachytene stage of meiotic prophase I. 10 ug of protein extracts was used per lane. Postnatal day 20 (PND20) wild type and *Scml2* ^-/Y^ testes serve as positive and negative controls respectively. ACTB serves as a loading control. Note that the abundance of SCML2 in the embryonic ovary is much lower than that in the testis. (B) Immunofluorescence analysis of SCML2 in spread nuclei of oocytes from E17.5 wild type ovaries. In contrast with the strong expression of SCML2 in male germ cells (Fig. 1E), SCML2 was not detected in spread nuclei of prophase I oocytes.
(TIF)

S2 Fig Schematic diagram of the *Scml2* targeting strategy. The mouse *Scml2* gene consists of 32 exons, based on the predicted cDNA sequence (XM_006528670.1). In the conditional *Scml2* ^fl^ allele, exon 11 is floxed. Exon 11 (244 bases; aa 279–360) encodes the second MBT repeat (aa 266–357 in Fig. 1A). Deletion of exon 11 is expected to cause a frame shift in the resulting *Scml2* mutant transcript. HyTK is a selection marker.
(TIF)

S3 Fig Specificity of anti-SCML2 antibodies. Testis sections from 2-month-old *Scml2* ^-/Y^ mice were immunostained with anti-SCML2 and γH2AX antibodies. Nuclei were stained with DAPI. In contrast with the strong SCML2 signal in germ cells in the wild type seminiferous tubules (Fig. 1D), no signal was detected in the germ cells in the *Scml2* ^-/Y^ tubules with our anti-SCML2 antibody, showing that our antibody is specific. The interstitial signal (green) is autofluorescence. Abbreviations: XY, XY body; Pa, pachytene spermatocyte; RS, round spermatid; ES, elongated spermatid. Scale bar, 50 μm.
(TIF)

S4 Fig Analysis of chromosomal synapsis and distribution of spermatocytes. Spread nuclei of spermatocytes from postnatal day 20 wild type and *Scml2* ^-/Y^ mice were immunostained with anti-SYCP1 and anti-SYCP2 antibodies. (A) Normal chromosomal synapsis in *Scml2* ^-/Y^ pachytene spermatocytes. 321 wild type and 335 *Scml2* ^-/Y^ pachytene spermatocytes were counted. Three mice per genotype were analyzed. 99% of both wild type and *Scml2* ^-/Y^ pachytene spermatocytes had normal synapsis. (B) Distribution of spermatocytes from wild type and *Scml2* ^-/Y^ mice. >200 spermatocytes from each mouse (three mice per genotype) were counted.
(TIF)

S5 Fig Distribution of γH2AX and SUMO1 in wild type and *Scml2* ^-/Y^ spermatocytes. Spread nuclei of spermatocytes from postnatal day 18 to 20 wild type and *Scml2* ^-/Y^ mice were immunostained with anti-SYCP2 and anti-γH2AX (A) or anti-SUMO1 (B) antibodies. Spermatocytes at the leptotene, zygotene, pachytene, and diplotene stages are shown.
(TIF)

S6 Fig MSCI is intact in *Scml2* ^-/Y^ spermatocytes. (A) Expression of PRAMEL3, an X-encoded protein, is not affected in *Scml2* ^-/Y^ testes. Testis sections from 2-month-old mice were immunostained with anti-PRAMEL3 antibody (green). Nuclei were stained with DAPI. Abbreviations: Pa, pachytene spermatocytes; RS, round spermatids. Scale bar, 50 μm. (B) Cot-1 RNA FISH of wild type and *Scml2* ^-/Y^ spermatocytes. The XY chromatin was positive for γH2AX but negative for Cot-1 in both wild type and *Scml2* ^-/Y^ spermatocytes. The panels were shown in views of Cot-1/γH2AX/DAPI, Cot-1/γH2AX, Cot-1 alone, and DAPI alone.
(TIF)
