# Supplementary figures and images for: Polycomb Protein SCML2 Associates with USP7 and Counteracts Histone H2A Ubiquitination in the XY Chromatin during Male Meiosis
Source: PLoS Genet. 2015 Jan 29;11(1):e1004954. doi: 10.1371/journal.pgen.1004954 (PMC4310598; doi:10.1371/journal.pgen.1004954)

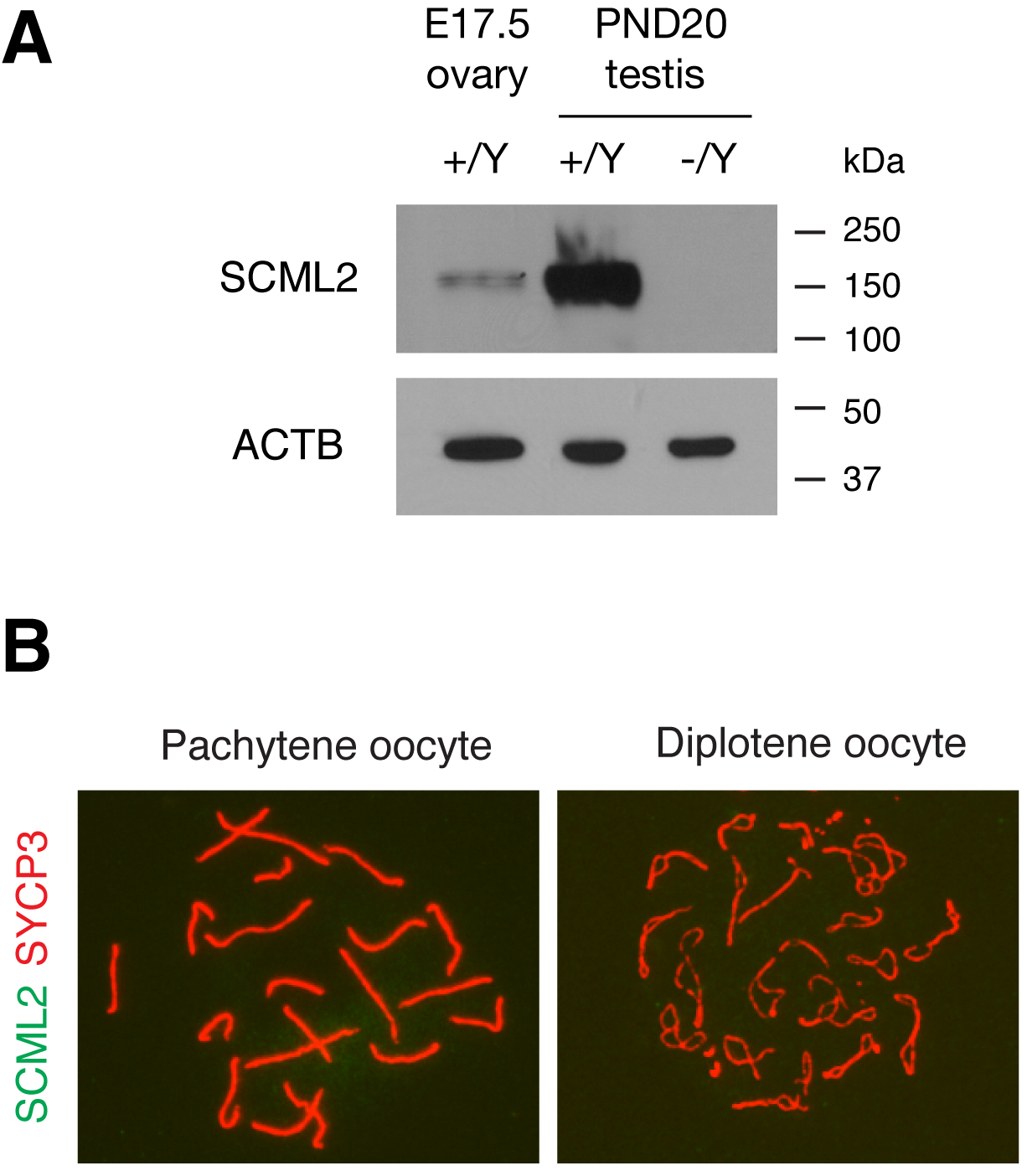

Supplement: S1 Fig — (A) Western blot analysis of SCML2 in embryonic day 17.5 (E17.5) ovaries. In E17.5 ovaries, most oocytes are at the pachytene stage of meiotic prophase I. 10 ug of protein extracts was used per lane. Postnatal day 20 (PND20) wild type and Scml2 -/Y testes serve as positive and negative controls respectively. ACTB serves as a loading control. Note that the abundance of SCML2 in the embryonic ovary is much lower than that in the testis. (B) Immunofluorescence analysis of SCML2 in spread nuclei of oocytes from E17.5 wild type ovaries. In contrast with the strong expression of SCML2 in male germ cells (Fig. 1E), SCML2 was not detected in spread nuclei of prophase I oocytes. (TIF) [file pgen.1004954.s001.tif]

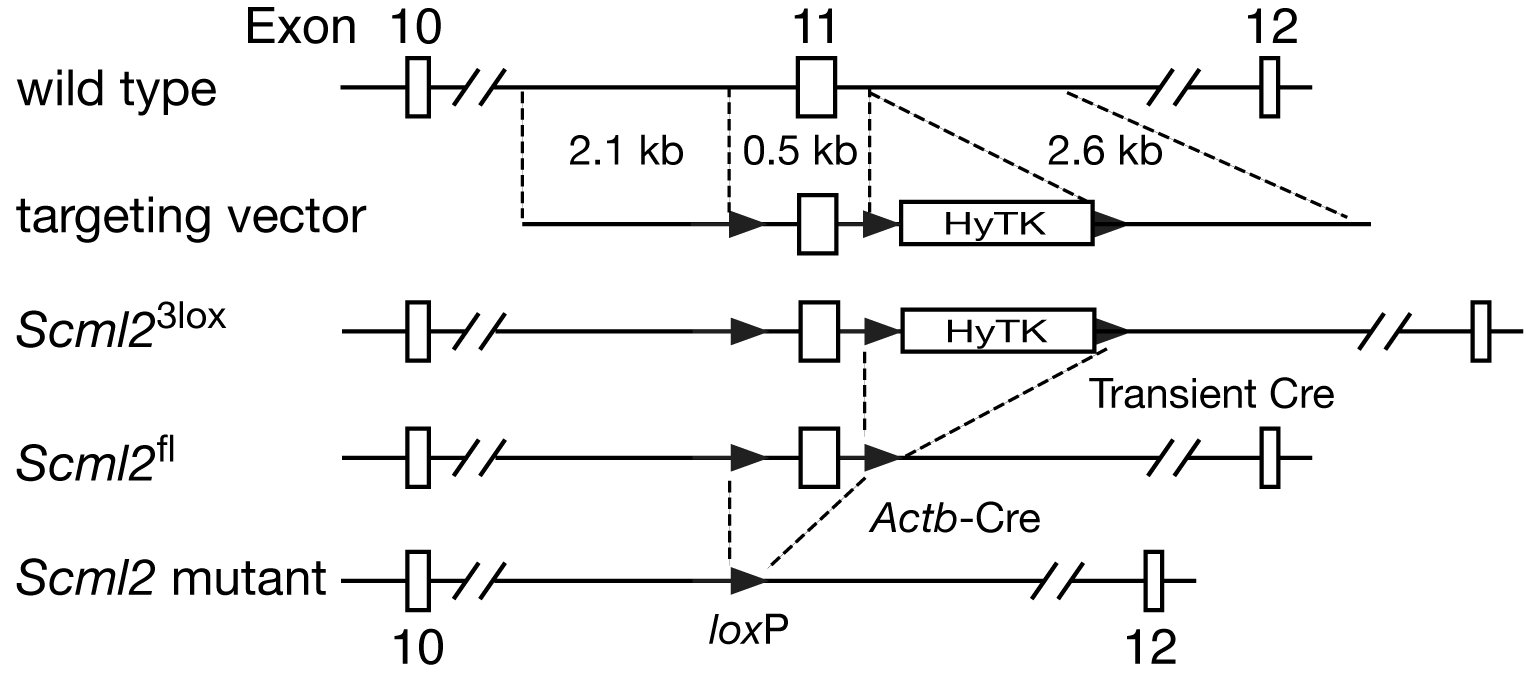

Supplement: S2 Fig — The mouse Scml2 gene consists of 32 exons, based on the predicted cDNA sequence (XM_006528670.1). In the conditional Scml2 fl allele, exon 11 is floxed. Exon 11 (244 bases; aa 279–360) encodes the second MBT repeat (aa 266–357 in Fig. 1A). Deletion of exon 11 is expected to cause a frame shift in the resulting Scml2 mutant transcript. HyTK is a selection marker. (TIF) [file pgen.1004954.s002.tif]

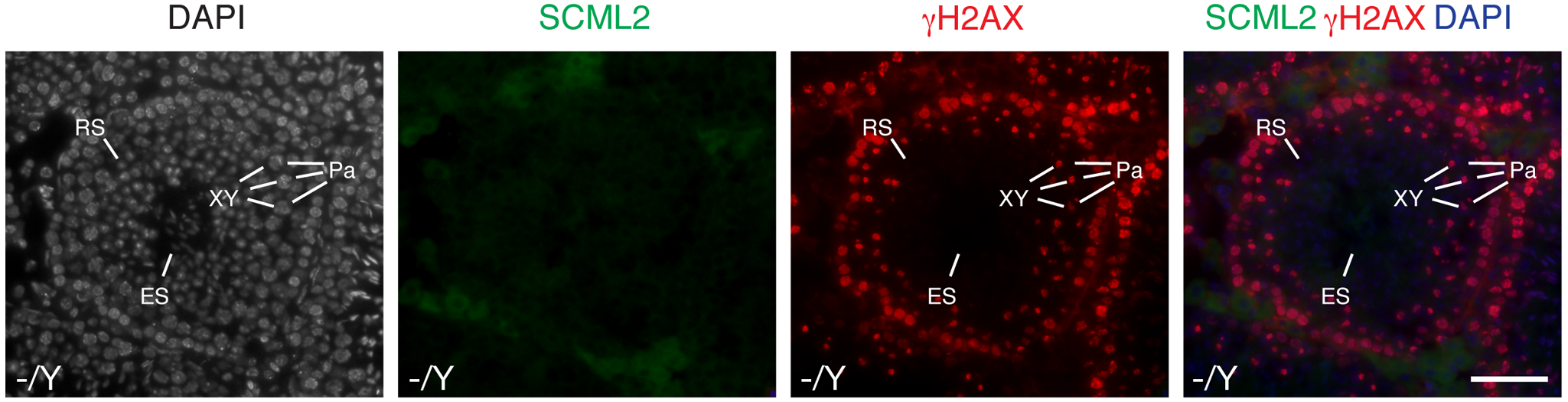

Supplement: S3 Fig — Testis sections from 2-month-old Scml2 -/Y mice were immunostained with anti-SCML2 and γH2AX antibodies. Nuclei were stained with DAPI. In contrast with the strong SCML2 signal in germ cells in the wild type seminiferous tubules (Fig. 1D), no signal was detected in the germ cells in the Scml2 -/Y tubules with our anti-SCML2 antibody, showing that our antibody is specific. The interstitial signal (green) is autofluorescence. Abbreviations: XY, XY body; Pa, pachytene spermatocyte; RS, round spermatid; ES, elongated spermatid. Scale bar, 50 μm. (TIF) [file pgen.1004954.s003.tif]

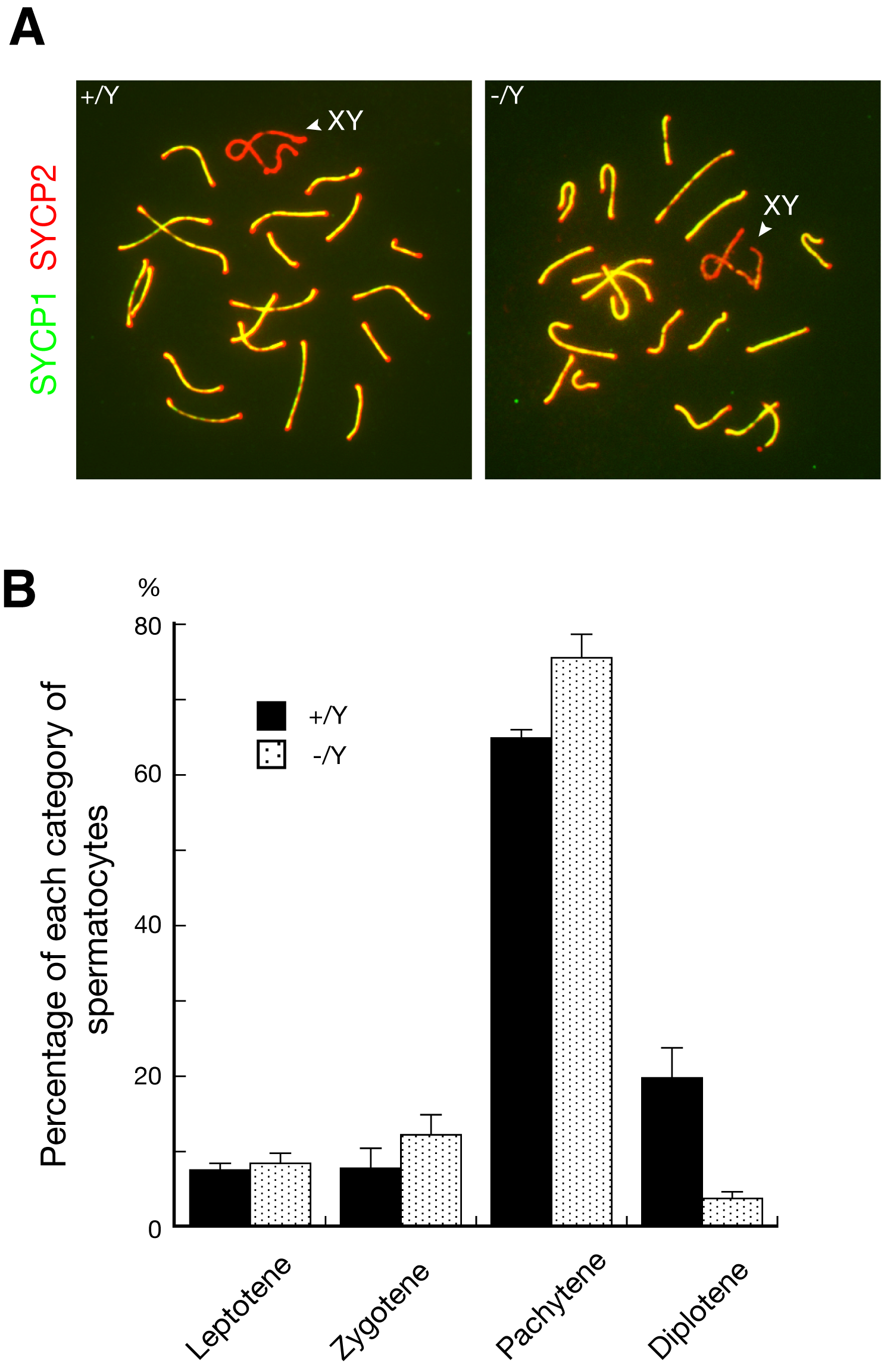

Supplement: S4 Fig — Spread nuclei of spermatocytes from postnatal day 20 wild type and Scml2 -/Y mice were immunostained with anti-SYCP1 and anti-SYCP2 antibodies. (A) Normal chromosomal synapsis in Scml2 -/Y pachytene spermatocytes. 321 wild type and 335 Scml2 -/Y pachytene spermatocytes were counted. Three mice per genotype were analyzed. 99% of both wild type and Scml2 -/Y pachytene spermatocytes had normal synapsis. (B) Distribution of spermatocytes from wild type and Scml2 -/Y mice. >200 spermatocytes from each mouse (three mice per genotype) were counted. (TIF) [file pgen.1004954.s004.tif]

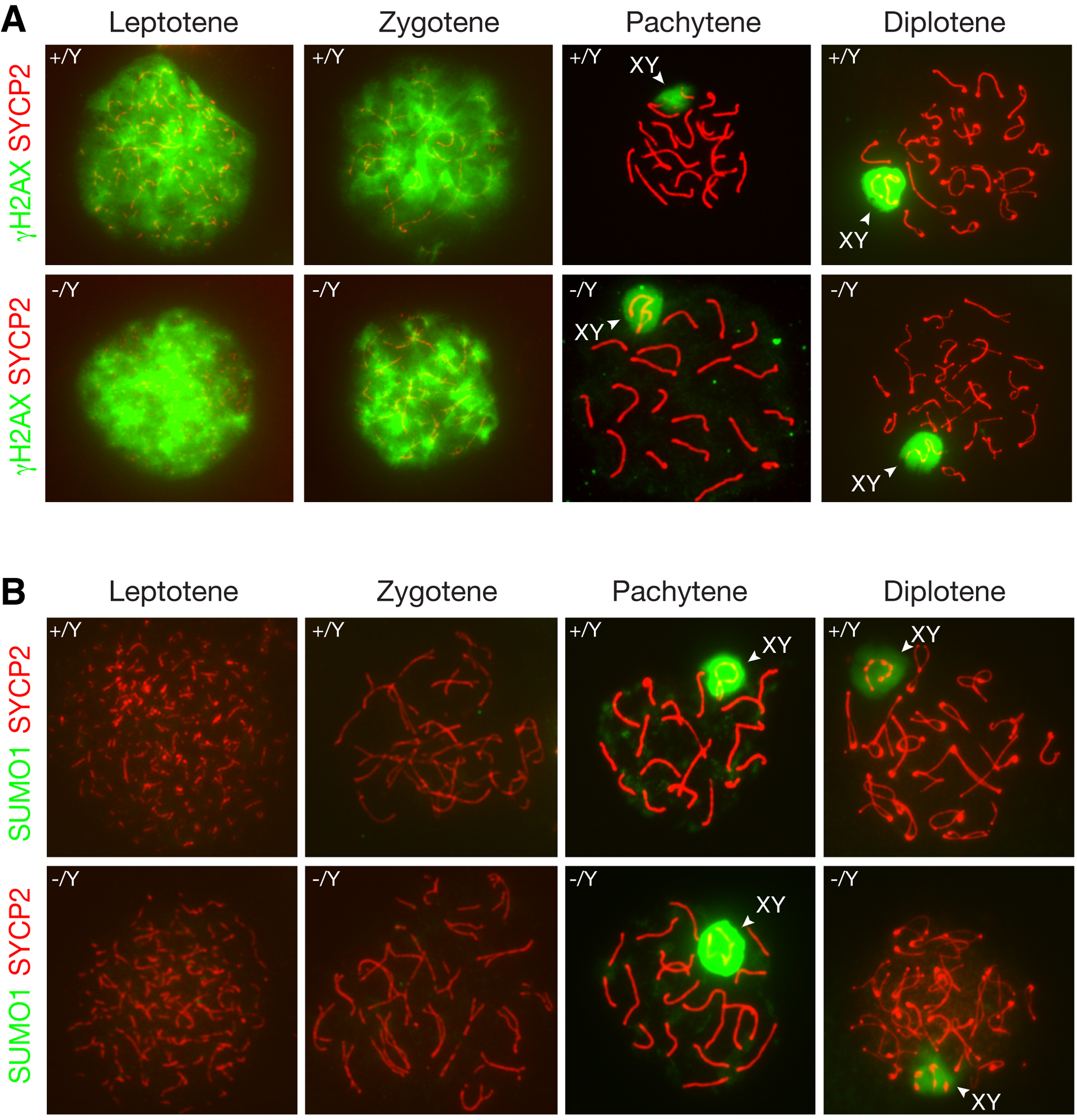

Supplement: S5 Fig — Spread nuclei of spermatocytes from postnatal day 18 to 20 wild type and Scml2 -/Y mice were immunostained with anti-SYCP2 and anti-γH2AX (A) or anti-SUMO1 (B) antibodies. Spermatocytes at the leptotene, zygotene, pachytene, and diplotene stages are shown. (TIF) [file pgen.1004954.s005.tif]

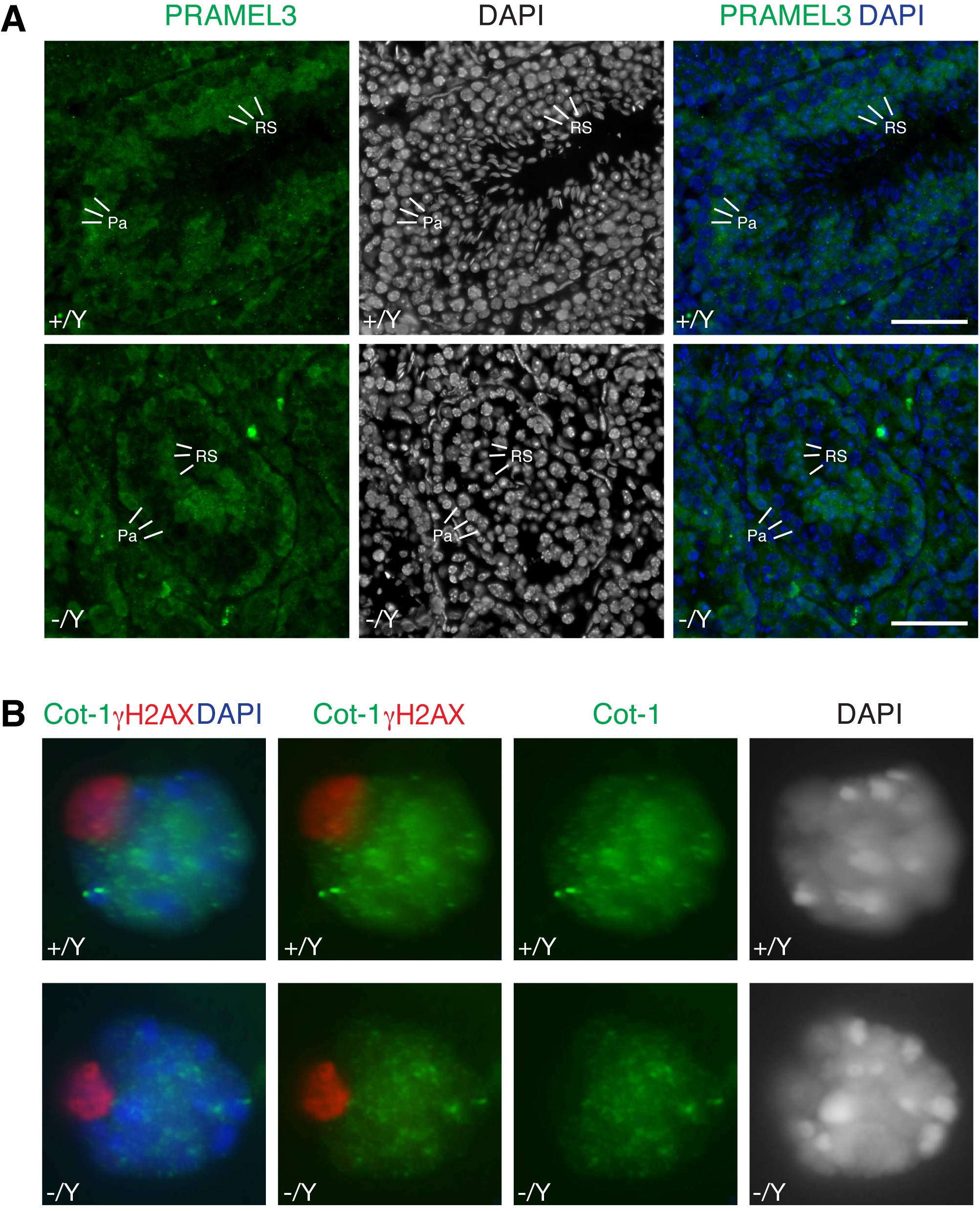

Supplement: S6 Fig — (A) Expression of PRAMEL3, an X-encoded protein, is not affected in Scml2 -/Y testes. Testis sections from 2-month-old mice were immunostained with anti-PRAMEL3 antibody (green). Nuclei were stained with DAPI. Abbreviations: Pa, pachytene spermatocytes; RS, round spermatids. Scale bar, 50 μm. (B) Cot-1 RNA FISH of wild type and Scml2 -/Y spermatocytes. The XY chromatin was positive for γH2AX but negative for Cot-1 in both wild type and Scml2 -/Y spermatocytes. The panels were shown in views of Cot-1/γH2AX/DAPI, Cot-1/γH2AX, Cot-1 alone, and DAPI alone. (TIF) [file pgen.1004954.s006.tif]
